# High mobility group box-1 levels in schizophrenia: Potential biomarker of remission phase

**DOI:** 10.5937/jomb0-28108

**Published:** 2021-06-05

**Authors:** Nuryil Yilmaz, Zekeriya Yelboga, Yavuz Yilmaz, Ozlem Demirpence

**Affiliations:** 1 Cumhuriyet University, Faculty of Medicine, Department of Psychiatry, Sivas, Turkey; 2 Cumhuriyet University, Faculty of Medicine, Department of Biochemistry, Sivas, Turkey

**Keywords:** schizophrenia, HMGB-1, remission and acute exacerbation phase, shizofrenija, HMGB-1, faza remisije i akutnog pogoršanja

## Abstract

**Background:** Schizophrenia is a chronic mental disorder, characterized byacute exacerbation and remission phases. Immune system has a role in the pathophysiology of schizophrenia. High mobility group box-1 (HMGB-1) is a macrophage secreted protein activating immune cells to produce cytokines. The aim of this study was to evaluate HMGB-1 levels among patients with schizophrenia both in acute exacerbation and remission phases.

**Methods: **Consecutive schizophrenia patients in acute exacerbation and remission phases were enrolled and compared with each other and with age-sex matched healthy subjects. Patients were assessed with the Scale for the Assessment of Positive Symptoms (SAPS), Scale for the Assessment of Negative Symptoms (SANS), Brief Psychiatric Rating Scale (BPRS), Clinical Global Impression Scale (CGI).

**Results: **Mean HMGB-1 levels were not significantly different in acute exacerbation phase versus remission phase schizophrenia patients (2.139±0.564 g/L vs. 2.326± 0.471 g/L, p=0.335) and both were individually higher than the control group (1.791±0.444 g/L, p=0.05 for acute exacerbation vs control, p=0.002 for remission vs control). In remission phase schizophrenic patients, HMGB-1 levels were positively correlated with Scale For The Assessment of Positive Symptoms (r=0.447, p=0.015) and BPRS (r=0.397, p=0.033) scores and HMGB-1 levels were independently associated with BPRS.

**Conclusions:** Serum HMGB-1 levels were shown to be increased in patients with schizophrenia patients irrespective of phase, there were no differences between patients in acute exacerbation and remission phase in terms of biomarker and HMGB-1 levels were related to symptom severity according to psychiatric scales among patients in remission phase of schizophrenia.

## Abstract (sr)

**Uvod:** Shizofrenija je hronični mentalni poremećaj koji karakterišu akutno pogoršanje i faze remisije. Imuni sistem ima ulogu u patopsihologiji shizofrenije. Protein HMGB-1 (eng. high mobility group box-1) je protein koji se stvara u makrofagima i aktivira imunoćelije da proizvode citokine. Cilj ove studije je da se procene nivoi proteina HMGB-1 kod pacijenata sa shizofrenijom i prilikom akutnog pogoršanja i u fazama remisije.

**Metode: **Obuhvaćeni su konsekutivni pacijenti sa shizofrenijom kod akutnog pogoršanja i u fazama remisije i upoređeni su jedni sa drugima i sa zdravim ispitanicima iste starosti i pola. Pacijenti su ispitani pomoću skale za procenu pozitivnih simptoma (SAPS), skale za procenu negativnih simptoma (SANS), kratke skale za psihijatrijsku procenu (BPRS) i globalne kliničke procene (CGI).

**Rezultati: **Srednji nivoi proteina HMGB-1 se nisu značajno razlikovali kod pacijenata u fazi akutnog pogoršanja u odnosu na pacijente u fazi remisije (2,139±0,564 g/L vs. 2,326±0,471 g/L, p=0,335) a obe grupe su imale značajno povećane nivoe od kontrolne grupe (1,791± 0,444 g/L, p=0,05 za akutno pogoršanje u odnosu na kontrolnu grupu, p=0,002 za fazu remisije u odnosu na kontrolnu grupu). Kod pacijenata sa shizofrenijom u fazi remisije, nivoi proteina HMGB-1 bili su u pozitivnoj korelaciji sa skalom za procenu pozitivnih simptoma (r=0,447, p=0,015), a rezultati procene BPRS (r=0,397, p=0,033) i nivoi proteina HMGB-1 bili su nezavisno povezani sa procenom BPRS.

**Zaključak:** Pokazalo se da su nivoi HMGB-1 u serumu povećani kod pacijenata sa shizofrenijom bez obzira na fazu i da su nivoi proteina HMGB-1 povezani sa ozbiljnošću simptoma prema psihijatrijskoj skali kod pacijenata u fazi remisije shizofrenije.

## Introduction

Schizophrenia is a chronic mental disorder with a heterogeneous presentations which include positive and negative symptoms in the form of hallucinations, delusions and negativism, and in addition to those, cognitive deficits [1]. Immune system represents one of the up-to-date interests in the pathophysiology of schizophrenia since immunological pathways might potentially be responsible for modulating the complex relation between environmental factors and genetic tendency [2] [3] [4] [5]. In a recent meta-analysis by Miller et al. [6] some cytokines; such as interleukin-12 (IL-12), IFN-γ, TNF-α, and sIL-2R levels were shown to be increased among different patient populations with schizophrenia in acute and remission phases, though, IL-1β, IL-6, and TGF-β levels were shown to be increased only during the acute exacerbation phase. Hence, there might be differential expression of pathways in two different phases of schizophrenia.

High mobility group box-1 (HMGB-1) proteinis a non-histone chromosomal protein with high electrophoretic mobility [7]. It was reported that HMGB-1 was released as a terminal inflammatory response, derived from necrotic cells or activated macrophages in response to organ failure [7]. HMGB-1 is described as a macrophage secreted protein that activated immune cells to produce cytokines [8].

HMGB-1 was shown to activate neutrophils to produce proinflammatory mediators such as TNF-α, L-1β and IL-8 [9]. On the other hand, HMGB-1 seems not only to play multiple roles in the pathogenesis of inflammatory pathways and autoimmune diseases but also to mediate renovation pathways in the body [10].

The aim of this study was first to check the association of HMGB-1 levels with two different phases of schizophrenia, i.e, acute exacerbation phase andremission phase and compare them with healthy controls (control group); and second to relate HMGB-1 to symptom scores,in relation to disease phase.

## Materials and Methods

In this cross-sectional study, consecutive schizophrenia patients in the acute exacerbation phase (Group 1) and remission phase (Group 2), who were admitted to Cumhuriyet University Hospital, Department of Psychiatry between July 2015 and December 2015, were included in the study and were compared with age-sex matched healthy subjects (Group 3) with no history of psychiatric disease in either themselves or in their families. Remission phase was described as schizophrenic patients in remission lasting at least 6 months at the time of giving consent to participate the study while being followed up in the outpatient department. Schizophrenic patients in the acute exacerbation phase were enrolled from Psychiatry ward within the first 3 days of hospitalization. Inclusion criteria: Being diagnosed according to the DSM-V diagnostic criteria by an expert psychiatrist along with persistence of psychotic symptoms of at least six months, being older than 18 years. Exclusion criteria: Having any infectious disease within the past month, using any medication that will affect the immune system or hormones, history of chronic inflammatory disease, absence of chronic psychiatric medical theray, history of organic mental disorder or mental retardation or an additional psychiatric disorder and patients with de novo disease (i.e, patient with first ever episode of disease).

Blood samples (5 mL) were obtained from the patients following interview. Serum samples were centrifuged at 1000 g for 15 minutes within 2 hours. These serum samples were immediately placed in ependoa and frozen at -80°C until the appropriate time for analysis. Serum HMGB-1 levels were assessed by quantitative sandwich enzyme-linked immunosorbent assay (ELISA) assays.

### Sociodemographic data form

The age, gender, smoking, alcohol, family life, self care, previous number of hospitalizations (index hospitalization of schizophrenic patients with acute exacerbation was not considered), duration of illness, family history of pychiatric disease, history of suicide attempt, current drug therapy were evaluated.

### Scale for the Assessment of Positive Symptoms (SAPS)

This scale is assessed by the interviewer, and is used tomeasure the level, distribution, and severity of positive symptoms of schizophrenia. It consists of 4 subscales and 34 items evaluating the evangelists, delusions, strange behavior and formal thought disorder. Each item has a score of 0 to 5, with a total score of 0–170. The scale was developed by Andreasen [11] and the adaptation of the Turkish form was made by Erkoç et al. (Erkoç et al., 1991, Düşünen Adam, not indexed in SCIE).

### Scale for the Assessment of Negative Symptoms (SANS)

This scale is a measure, assessed by the interviewer, and is used to measure the level, distribution and severity of negative symptoms of schizophrenia. It consists of 5 subscales and 25 items evaluating affective blunting, aloji, apathy, anhedonia and attention deficit. Each item has a score of 0 to 5, with a total score of 0–125. The scale was developed by Andreasen [11] and the adaptation of the Turkish version was made by Erkoç et al. (Erkoç et al., 1991, Düşünen Adam, not indexed in SCIE).

### Brief Psychiatric Rating Scale (BPRS)

This scale was developed by Overall [12]. BPRS is used in psychiatric patient groups to measure the change in pharmacological treatment. It is a measure of symptom severity (0 = absent, 6 = very severe) consisting of 24 items and each item is rated. The score can range from 0 to 144.

### Clinical Global Impression Scale (CGI)

This was developed and is designed to enable a clinician to record the impression of a patient's function before and after initiation of treatment [13]. In the first dimension of the scale, the severity of the disease, in the second dimension, the improvement, and in the third dimension,the severity of the side effect is evaluated.

This study was approved by the Clinical Trials Ethics Committee of Cumhuriyet University of Faculty of Medicine (Date: 14.07.2015 and number: 2015-07/02).

### Statistical analysis

All data were recorded and then evaluated by SPSS 22.0, a registered institutional software. Parametric data for comparison of three initial subgroups were evaluated by ANOVA test with post hoc comparison of Tukey's HSD. Comparison of schizophrenic patients in acute exacerbation phase and 2 for further analysis (schizophrenic scales) was provided via independent sample t test. Tests for homogeneity of variances were provided via Levene statistics in both occasions. Categorical data were evaluated by appro priate chi square testing. Correlation was evaluated via Pearson's correlation test. A linear regression analysis was provided to predict Brief Psychiatric Rating Scale. A p value 0.05 was accepted as significant.

## Results

There were 30 patients (10/20 Female/Male) in Group 1 (schizophrenic patients in acute exacerbation phase), 29 patients (9/20 Females/Males) in Group 2 (schizophrenic patients in remission phase) and 15 healthy age-sex matched individuals in Group 3 (6/9, Females/Males). Mean age of the Group 1, Group 2 and Group 3 were similar to each other (37.5±11.4 vs 38.5±11 vs 33.3±8.2 years, p=0.298 for Anova, p=0.074 for homogeneity of variances test result), and, individual post-hoc comparisons yielded no significant difference of both patient groups from each other and the control group (P=0.938 for Group 1 vs Group 2, p=0.420 for Group 1 vs Group 3 and p=0.280 for Group 2 vs Group 3). Of note, in the whole cohort (n=74), overall, age was not correlated with serum HMGB-1 levels (r=-0.164, p=0.164).

There was no statistically significant difference between the two schizophrenia subgroups (Group 1 and 2) in terms of smoking, alcohol, living with family, inability to self care, family history of pychiatric disease, history of suicide attempt, antipsychotic medication, disease duration (years), number of previous hospitalizations (Table 1).

**Table 1:** Comparison of basal characteristics in two groups F/M: Female/Male, SAPS: Scale for the Assessment of Positive Symptoms SANS: Scale for the Assessment of Negative Symptoms, BPRS: Brief Psychiatric Rating Scale CGI: Clinical Global Impression Scale

	Schizophrenic patients in acute exacerbation phase (n=30)	Schizophrenic patients in remission phase (n=29)	P for dual comparison
Age	37.5±11.4	38.5±11	<0.938
Gender (F/M)	10/20	9/20	0.159
Smoking	13/30	11/29	0.673
Alcohol	26/30	22/29	0.465
Living with family	26/30	24/29	0.731
Inability to self care	22/30	16/29	0.236
Family history of pychiatric disease	12/30	9/29	0.655
History of suicide attempt	6/30	4/29	0.731
Typical Antipsychotic Atypical Antipsychotic Typical + Atypical Antipsychotic Typical Antipsychotic + mood stabilizers Atypical antipsychotic+ mood stabilizers	3/22/3/0/2	1/23/3/1/1	0.674
Duration of illness (years)	10.5±8.5	10.7	0.755
Previous number of hospitalization	4.4±4.4 (excluding index hospitalization)	3.9±2.8	0.652
HMGB-1 (μg/L)	2.139±0.564	2.326±0.471	0.335
SAPS	29.2±13.5	5±4.7	<0.001*
SANS	29.4±23.9	8.9±7.9	<0.001*
BPRS	22.6±10.3	4.1±3.9	<0.001*
CGI	8.4±1.2	6.5±1.9	<0.001*

**Figure 1:**
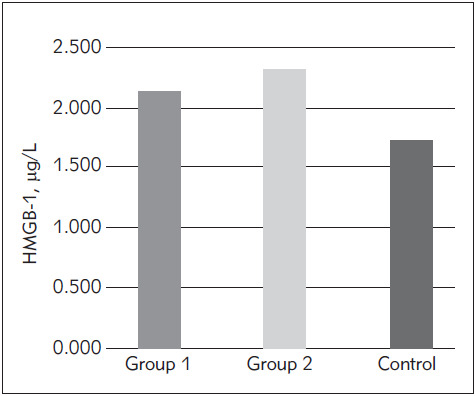
HMGB-1 levels in schizophrenia patients with acute exacerbation versus remission versus healthy controls

Mean serum HMGB-1 levels were 2.139±0.564 μg/L, 2.326±0.471 μg/L and 1.791±0.444 μg/L for Group 1, 2 and 3 respectively (p=0.004 for Anova, p=0.501 for homogeneity of variances test result) (Figure 1). Mean levels of HMGB-1 in Group 1 and 2 were similar and both were significantly higher than those in control group (p=0.335 Group 1 and 2, p=0.05 for Group 1 vs Group 3, p=0.002 for Group 2 vs Group 3).

In comparison of Group 1 and 2 with regard to tested scores, there were significant differences (Table 2). In Group 1, serum HMGB-1 levels were not correlated with SAPS, SANS, BPRS, CGI scores. However, in Group 2, HMGB-1 levels were moderately and positively correlated with both SAPS (r=0.447, p=0.015) and BPRS (r=0.397, p=0.033) scores. In the remission phase of schizophrenia patients (Group 2), age, duration of illness, number of previous hospitalizations and HMGB-1 levels were enrolled into regression analysis and it was found that HMGB-1 levels were independently associated with BPRS in linear regression analysis (Table 3).

**Table 2:** Correlation of HMGB-1 levels with demographic and schizophrenia scores

	SAPS	SANS	BPRS	CGI	Age	Disease duration (years)
Group 1 (n=30)						
HMGB-1 (µg/L)	r=-0.129 p=0.498	r=-0.060 p=0.751	r=-0.104 p=0.585	r=-0.299 p=0.108	r= -0.348 p=0.059	r=-0.239 p=0.204
Group 2 (n=29)						
HMGB-1 (µg/L)	r=0.447 p=0.015*	r=0.301 p=0.113	r=0.397 p=0.033*	r=0.124 p=0.521	r=-0.237 P=0.217	r=-0.011 p=0.954

**Table 3:** Linear regression analysis to predict »Brief Psychiatric Scale« score in remission phase of schizohprenia patients

	B	S.E.	Wald	df	Sig.	Exp(B)	95% C.I.for EXP(B)	
							Lower	Upper
HMGB-1	0.002	0.001	9.639	1	0.002	1.002	1.001	1.003
Constant	-2.709	1.107	5.990	1	0.014	1.067		

## Discussion

Relationship between inflammation and schizophrenia has been investigated in a sizable number of studies before in the literature [1] [2] [4] [5]. Many interleukins have been studied in schizophrenia [14] [15] [16].

In this study, HMGB-1, as an inflammatory marker, was evaluated in patients suffering from acute exacerbation and in patients with chronic remission of schizophrenia. Serum HMGB-1 levels of of Group 1 and 2 were similar and both levels were significantly higher than the healthy control, Group 3. In one study from China, the serum levels of HMGB-1, IL-10, IL-6 and TNF-α in schizophrenic patients were reported to be significantly higher in the healthy controls [17]. After treatment with risperidone for 6 months, the serum levels of HMGB-1, L-1β, TNF-α and IL-6 were reported to be decreased. In some of the studies, the levels of IL-12p40, IL-3 in chronic schizophrenic patients were found to be significantly higher than healthy controls [14] [15]. In a study by Borovcanin et al. [16] it was reported that serum levels of IL-23 were elevated in all clinical phases of schizophrenia, independent from the treatment. In our study, mean age of the Group 1, Group 2 and Group 3 were not significantly different from each other. Besides, in thewhole cohort (n=74) age was not correlated with HMGB-1 levels. Furthermore, in a recent study about cytokine levels in schizophrenia, age was not related to cytokine levels and did not differ between diagnostic phenotypes [18].

Confirming previous studies regarding cytokine levels, there was no statistically significant association between HMGB-1 levels and duration of illness (years) and also antipsychotic therapy [14] [15] [18] [19]. Of note, in the study of Zhu et al. [17] IL-6 and HMGB-1 levels and in the study of Borovcanin et al. [20] IL-6 levels were shown to decrease following therapy. Different immunological processes may potentially take place during the different phases of schizophrenia. Of note, HMGB-1 is known to be released by two distinct pathways, either by non-apoptotic cell death or by active secretion of innate immune cells, mainly microglia cell, particularly in the hippocampal region immediatey after stress exposure. Hence, HMGB-1 might have a role in stress-induced neuroinflammatory response [21] [22], though, in our study, we did not observe any finding specific to acute exacerbation phase and HMGB-1 levels were rather reflective of findings in chronic remission phase. In remission phase, HMGB-1 levels were positively correlated with both SAPS and BPRS scores. Hence, in chronic phase, as the positive symptom severity increased, levels of HMGB-1 also increased further adding the findings of a recent study, which showed that patients with more severe positive symptoms had higher IL-6 levels [18].

There are some limitations of the current study worthwhile mentioning. Sample size was relatively small and all patients were from a single tertiary care center. Hence, the results can not be generalized to all phenotypes of schizophrenia. As, patients with de novo disease, i.e, hospitalized schizophrenic patients who had their diagnosis at that index admission were not included in this study. Besides, all patients irrespective of phase of schizophrenia were those who had been treated with antipsychotic medications according to the discretion of their primary physician. Hence, potential influence of antipsychotic medications on HMGB-1 levels remains unanswered, though, the two groups were similar with regard to type of antipsychotic therapy. Some potentially confounding factors such as body mass index (BMI) and other sources of stress, which may potentially affect the cytokine levels were not fully considered in this study [6] [15]. Of note, none of the patients had BMI>30 kg/m^2^ in the study. Furthermore, this study might not give a final answer that HMGB-1 is a state or trait marker. Of note, all schizophrenic patients were on chronic antipsychotic medications in this study, since deprescribing the patients' antipsychotic therapy for research was not found ethically acceptable. Hence, in order to precisely delineated the temporal relationship of HMGB-1 with the symptoms of schizophrenia, blood samples should be obtained from drug-naive patients with schizophrenia in the first-episode on longitudinal studies.

## Conclusions

Serum HMGB-1 levels were shown to be increased in patients with schizophrenia with or without acute exacerbation and higher in patients with schizohprenia compared to healthy controls, and hence, do not differ significantly in remission phase versus acute exacerbation. On the other hand, HMGB-1 levels seem to be positively correlated with symptom severity scores in patients with remission phase of schizophrenia, whereas, the biomarker levels do not relate to symptom scores in acute exacerbation phase. Therefore, it seems that HMGB-1 might potentially be a marker of chronic disease status of schizophrenia, though it remains to be established in larger longitudinal cohorts in relation to therapy.

In conclusion, to the best of our knowledge, we show for the first time in the literature that serum HMGB-1 levels reflect the status of patients with schizophrenia in chronic remission phase rather than acute exacerbations, and are associated with disease severity scores among these patients in remission phase.

## Acknowledgments

None.

## Conflict of interest statement

All the authors declare that they have no conflict of interest in this work.

## References

[1] Na K S, Jung H Y, Kim Y K (2014). The role of pro-inflammatory cytokines in the neuroinflammation and neurogenesis of schizophrenia. Prog Neuropsychopharmacol Biol Psychiatry.

[2] Ozbey U, Tug E, Kara M, Namli M (2008). The value of interleukin-12B (p40) gene promoter polymorphism in patients with schizophrenia in a region of East Turkey. Psychiatry Clin Neurosci.

[3] Watanabe Y, Someya T, Nawa H (2010). Cytokine hypothesis of schizophrenia pathogenesis: Evidence from human studies and animal models. Psychiatry Clin Neurosci.

[4] Altamura C A, Pozzoli S, Fiorentini A, Dell'osso B (2013). Neurodevelopment and inflammatory patterns in schizophrenia in relation to pathophysiology. Prog Neuropsychopharmacol Biol Psychiatry.

[5] García-Bueno B, Bioque M, Mac-Dowell K S, Barcones F M, Martínez-Cengotitabengoa M, Pina-Camacho L, Rodríguez-Jiménez R, Sáiz P A, Castro C, Lafuente A, Santabárbara J, González-Pinto A (2014). Pro-/Anti-inflammatory Dysregulation in Patients With First Episode of Psychosis: Toward an Integrative Inflammatory Hypothesis of Schizophrenia. Schizophr Bull.

[6] Miller B J, Buckley P, Seabolt W, Mellor A, Kirkpatrick B (2011). Meta-Analysis of Cytokine Alterations in Schizophrenia: Clinical Status and Antipsychotic Effects. Biol Psychiatry.

[7] Yamada S, Maruyama I (2007). HMGB1, a novel inflammatory cytokine. Clin Chim Acta.

[8] Yang H, Tracey K J (2010). Targeting HMGB1 in inflammation. Biochim Biophys Acta Gene Regul Mech.

[9] Park J S, Arcaroli J, Yum H K, Yang H, Wang H, Yang K, Choe K, Strassheim D, Pitts T M, Tracey K J, Abraham E (2003). Activation of gene expression in human neutrophils by high mobility group box 1 protein. Am J Physiol Cell Physiol.

[10] Magna M, Pisetsky D S (2014). The Role of HMGB1 in the Pathogenesis of Inflammatory and Autoimmune Diseases. Mol Med.

[11] Andreasen N C (1990). Methods for Assessing Positive and Negative Symptoms. Mod Probl Pharmacopsychiatry.

[12] Overall J E, Gorham D R (1988). The Brief Psychiatric Rating Scale BPRS: Recent developments in ascertainment and scaling: Introduction. Psychopharmacol Bull.

[13] Beneke M, Rasmus W (1992). 'Clinical Global Impressions' (ECDEU): Some Critical Comments. Pharmacopsychiatry.

[14] Bedrossian N, Haidar M, Fares J, Kobeissy F H, Fares Y (2016). Inflammation and Elevation of Interleukin-12p40 in Patients with Schizophrenia. Front Mol Neurosci.

[15] Xiu M H, Lin C G, Tian L, Tan Y L, Chen J, Chen S, Tan S P, Wang Z R, Yang F D, Chen D C, Zhang X Y (2015). Increased IL-3 serum levels in chronic patients with schizophrenia: Associated with psychopathology. Psychiatry Res.

[16] Borovčanin M, Jovanović I, Đukić-Dejanović S, Radosavljević G, Arsenijević N, Lukić M L (2015). Increase systemic levels of IL-23 as a possible constitutive marker in schizophrenia. Psychoneuroendocrinology.

[17] Zhu Q, Li X, Hie G, Yuan X, Lu L, Song X (2015). Analysis of the changes of serum high mobility group protein B1 and cytokines in first-episode schizophrenia patients. Zhonghua Yi Xue Za Zhi (Taipei).

[18] Lee E E, Hong S Z, Martin A S, Eyler L T, Jeste D V (2017). Inflammation in Schizophrenia: Cytokine Levels and Their Relationships to Demographic and Clinical Variables. Am J Geriatr Psychiatry.

[19] Stojanović A, Martorell L, Montalvo I, Ortega L, Monseny R, Vilella E, Labad J (2014). Increased serum interleukin-6 levels in early stages of psychosis: Associations with at-risk mental states and the severity of psychotic symptoms. Psychoneuroendocrinology.

[20] Borovčanin M, Jovanović I, Radosavljević G, Đukić-Dejanović S, Stefanović V, Arsenijević N, Lukić M L (2013). Antipsychotics can modulate the cytokine profile in schizophrenia: Attenuation of the type-2 inflammatory response. Schizophr Res.

[21] Weber M D, Frank M G, Tracey K J, Watkins L R, Maier S F (2015). Stress Induces the Danger-Associated Molecular Pattern HMGB-1 in the Hippocampus of Male Sprague Dawley Rats: A Priming Stimulus of Microglia and the NLRP3 Inflammasome. J Neurosci.

[22] Yao X, Jiang Q, Ding W, Yue P, Wang J, Zhao K, Zhang H (2019). Interleukin 4 inhibits high mobility group box-1 protein-mediated NLRP3 inflammasome formation by activating peroxisome proliferator-activated receptor-g in astrocytes. Biochem Biophys Res Commun.

